# Acute-Phase Serum Amyloid A: An Inflammatory Adipokine and Potential Link between Obesity and Its Metabolic Complications

**DOI:** 10.1371/journal.pmed.0030287

**Published:** 2006-06-06

**Authors:** Rong-Ze Yang, Mi-Jeong Lee, Hong Hu, Toni I Pollin, Alice S Ryan, Barbara J Nicklas, Soren Snitker, Richard B Horenstein, Kristen Hull, Nelson H Goldberg, Andrew P Goldberg, Alan R Shuldiner, Susan K Fried, Da-Wei Gong

**Affiliations:** 1Division of Endocrinology, Diabetes, and Nutrition, University of Maryland School of Medicine, Baltimore, Maryland, United States of America; **2**Division of Gerontology, Geriatric Research, Education, and Clinical Center, Baltimore Veterans Administration Medical Center, Baltimore, Maryland, United States of America; **3**Section on Gerontology and Geriatric Medicine, Wake Forest University School of Medicine, Winston-Salem, North Carolina, United States of America; **4**Division of Plastic and Reconstructive Surgery, University of Maryland School of Medicine, Baltimore, Maryland, United States of America; University of Pennsylvania School of MedicineUnited States of America

## Abstract

**Background:**

Obesity is associated with low-grade chronic inflammation, and serum markers of inflammation are independent risk factors for cardiovascular disease (CVD). However, the molecular and cellular mechanisms that link obesity to chronic inflammation and CVD are poorly understood.

**Methods and Findings:**

Acute-phase serum amyloid A (A-SAA) mRNA levels, and A-SAA adipose secretion and serum levels were measured in obese and nonobese individuals, obese participants who underwent weight-loss, and persons treated with the insulin sensitizer rosiglitazone. Inflammation-eliciting activity of A-SAA was investigated in human adipose stromal vascular cells, coronary vascular endothelial cells and a murine monocyte cell line. We demonstrate that A-SAA was highly and selectively expressed in human adipocytes. Moreover, A-SAA mRNA levels and A-SAA secretion from adipose tissue were significantly correlated with body mass index ( *r* = 0.47; *p* = 0.028 and *r* = 0.80; *p* = 0.0002, respectively). Serum A-SAA levels decreased significantly after weight loss in obese participants ( *p* = 0.006), as well as in those treated with rosiglitazone ( *p* = 0.033). The magnitude of the improvement in insulin sensitivity after weight loss was significantly correlated with decreases in serum A-SAA ( *r* = −0.74; *p* = 0.034). SAA treatment of vascular endothelial cells and monocytes markedly increased the production of inflammatory cytokines, e.g., interleukin (IL)-6, IL-8, tumor necrosis factor alpha, and monocyte chemoattractant protein-1. In addition, SAA increased basal lipolysis in adipose tissue culture by 47%.

**Conclusions:**

A-SAA is a proinflammatory and lipolytic adipokine in humans. The increased expression of A-SAA by adipocytes in obesity suggests that it may play a critical role in local and systemic inflammation and free fatty acid production and could be a direct link between obesity and its comorbidities, such as insulin resistance and atherosclerosis. Accordingly, improvements in systemic inflammation and insulin resistance with weight loss and rosiglitazone therapy may in part be mediated by decreases in adipocyte A-SAA production.

## Editors' Summary

**Background.:**

Obesity often alters an individual's overall metabolism, which in turn leads to complications like diabetes, high blood pressure, and an increased risk of cardiovascular disease (disease of the heart and blood vessels, such as stroke or heart attacks). Having established a strong link between inflammation and cardiovascular disease, scientists now think that obesity might cause persistent low-level inflammation, and that this is the reason for the cardiovascular problems seen in many obese people. By better understanding the links between obesity, inflammation, and cardiovascular disease, the hope is that scientists may be able to find medications that can be given to obese people to reduce their risk of heart attacks and strokes.

**Why Was This Study Done?:**

Previous research had suggested that a substance in the blood called A-SAA, which is raised by inflammation, might be a “missing link” between inflammation and cardiovascular disease, since an individual's baseline level of A-SAA is associated with the risk for cardiovascular disease (in other words, the higher the A-SAA, the higher the risk of cardiovascular disease). In the new study, researchers wanted to know whether the reason that obese people have a higher risk of cardiovascular disease is because they have higher blood levels of A-SAA.

**What Did the Researchers Do and Find?:**

They found that obese people had higher levels of A-SAA in their blood. A-SAA appears to be produced in fat cells (or adipocytes) and then released into the blood. Obese people have higher numbers of fat cells, which could by itself account for the higher blood levels of A-SAA, but the researchers also found that the average fat cell from an obese individual produces and secretes higher levels of A-SAA than fat cells from lean individuals. When the researchers studied people who underwent weight loss, they found that A-SAA levels fell in response to weight loss, and this was associated with improvements in their metabolism. They then studied obese individuals who received the diabetes drug rosiglitazone (which is known to reduce inflammation). They found that even though these individuals did not lose weight, their A-SAA levels dropped as their metabolism improved. Trying to get at the mechanisms by which A-SAA might cause inflammation and diabetes, the researchers found that exposure to A-SAA can stimulate the activation of proinflammation molecules in a number of different cells, including blood vessel cells. It can also stimulate cells to break down fat stores and release fats, which could lead to metabolic complications and ultimately contribute to diabetes.

**What Do These Findings Mean?:**

Together with similar results from other studies, the findings here suggest that A-SAA could promote inflammation, and that elevated levels of A-SAA in obese individuals could contribute to the chronic low-level inflammatory state that puts them at higher risk for cardiovascular complications. The authors speculate that drugs that reduce the blood levels of A-SAA might be useful as treatments for obese patients (to lower their risk of heart attacks and strokes). However, as they acknowledge, additional studies are needed to establish that A-SAA is indeed a causal link between obesity and inflammation and whether it plays a major role before it could be considered a promising drug target.

**Additional Information.:**

Please access these Web sites via the online version of this summary at http://dx.doi.org/10.1371/journal.pmed.0030287.

• MedlinePlus pages on obesity and cardiovascular disease

• US Centers for Disease Control and Prevention pages on obesity and cardiovascular disease

• Wikipedia pages on obesity and cardiovascular disease (note: Wikipedia is a free Internet encyclopedia that anyone can edit)

## Abstract 3

Higher levels of Acute-phase serum amyloid A (A-SAA), a proinflammatory adipokine, in obese individuals may contribute to the chronic low-level inflammatory state that puts them at higher risk for cardiovascular complications.

## Introduction

Complications of excess fat mass, particularly central or visceral adipose tissue, include insulin resistance and resulting hyperinsulinemia, glucose intolerance and diabetes, hypertension, hyperlipidemia, and a prothrombotic state. This constellation of obesity-related complications, often referred to as the metabolic syndrome or syndrome X [ 1, 2], markedly increases risk of cardiovascular disease (CVD) and death [ 3, 4].

Although the link between excess body fat and the metabolic and cardiovascular sequelae is well documented clinically and epidemiologically, the molecular and cellular underpinnings for this link are poorly understood. Excess and/or dysfunctional adipose tissue is associated with chronic low-grade systemic inflammation, which is also associated with CVD. For example, a modest elevation in C-reactive protein (CRP), an acute-phase reactant protein produced by the liver and a long-known marker of inflammation, has been shown to be predictive of CVD risk and events [ 5]. Indeed, measurement of CRP is now recommended in some clinical settings to stratify individual CVD risk and to help direct therapy [ 3, 6]. Serum amyloid A (SAA), another acute-phase reactant protein, has also been shown to be a predictor of CVD [ 7, 8]. Whether these acute-phase reactant proteins are directly involved in inflammation and the atherosclerotic process or simply markers of these processes is not known.

Adipose tissue is “inflamed” in obesity, with decreased expression of the anti-inflammatory adipokine adiponectin and increased secretion of a variety of proinflammatory cytokines, e.g., tumor necrosis factor alpha (TNF-α), interleukin (IL)-6, and prothrombotic factors such as plasminogen activator inhibitor-1 (PAI-1) [ 9]. Infiltration of adipose tissue by macrophages is in part responsible for this inflammatory process associated with obesity [ 10, 11]. However, the upstream regulator(s) responsible for the inflammatory state in adipose tissue and the role adipose tissue-derived inflammatory factors play in systemic inflammation remain unclear [ 12]. In a systematic search of differentially expressed genes between adipocytes and stromal vascular cells, we noted that serum amyloid A1 and A2 (SAA1 and SAA2, collectively called A-SAA) were highly expressed in human adipocytes, which was unexpected but in agreement with recent publications [ 13, 14]. A-SAA has been regarded as merely an inflammation marker and thought to be produced primarily in liver. In this study, we aimed to test the hypothesis that adipose A-SAA may be a molecular link between obesity and its comorbidities in humans.

## Methods

### Human Participants

The Institutional Review Boards of the respective institutions approved all human studies, and each volunteer provided written informed consent to participate. All participants were healthy according to medical history, physical examination, and laboratory testing unless otherwise specified in the protocols. The individuals studied showed no clinical or laboratory evidence of acute inflammation such as fever or elevated white blood cell counts. Abdominal adipose tissue samples were obtained from overnight-fasted participants by aspiration with a 4-mm cannula under local anesthesia with lidocaine as previously described [ 19] or obtained from nondiabetic participants undergoing intra-abdominal surgeries. All blood samples were stored at −80 °C until used.

#### Cross-sectional study of body mass index and serum A-SAA levels.

Participants were part of the previously described Amish Family Diabetes Study [ 20]. Initially, A-SAA levels were measured in plasma samples from 19 sex- and age-matched (age within 5 y) sets of nondiabetic sibling pairs with a discordance in body mass index (BMI) of at least 3 kg m ^−2^. These 38 individuals were then included in an expanded set of 134 nondiabetic individuals with BMIs ranging from 17.0 to 41.8 kg m ^−2^. Blood samples for A-SAA measurements were obtained from an antecubital vein after an overnight fast.

#### Effect of weight loss on A-SAA and insulin sensitivity.

Thirty-three sedentary, overweight or obese (BMI 32.3 ± 4.0 kg m ^−2^, mean ± standard deviation [SD]), postmenopausal (58 ± 5.7 y, mean ± SD) women were studied before and after a 6-mo weight loss program. The intervention consisted of weekly outpatient classes with a dietitian on the principles of a hypocaloric dietary program that followed the American Heart Association (AHA) Step I guidelines with restriction of caloric intake by 250–350 kcal d ^−1^ as previously described [ 21]. Fat mass was determined by dual-energy X-ray absorptiometry (Model DPX-L; Lunar Radiation, Madison, Wisconsin, United States) using the 1.3z DPX-L extended analysis program. Fasting serum levels of A-SAA were measured before and after the intervention in all participants. Subcutaneous abdominal fat biopsies were obtained at baseline in a subset of 31 participants under local anesthesia for the ex vivo studies of adipose A-SAA expression and/or secretion (described below). Eight of the participants underwent 3-h hyperinsulinemic-euglycemic clamp studies at baseline and after completion of the weight loss program to assess changes in insulin sensitivity. Women were weight-stabilized (< 1 kg) for at least 2 wk prior to metabolic testing before and after weight loss. All testing was performed in the morning after a 12-h overnight fast. Arterialized blood was obtained from a dorsal heated hand vein. Basal plasma glucose and insulin levels were measured in the fasted state. A 10 min priming with insulin followed by a continuous intravenous infusion of insulin (240 pmol m ^−2^ min ^−1^, Humulin, Eli Lilly, Indianapolis, Indiana, United States) was performed for 180 min. Glucose was infused at a variable rate to keep the plasma glucose, measured every 5 min, at the basal (fasting) level. Blood was also drawn every 10 min for the determination of plasma insulin levels. Plasma glucose was measured with the glucose oxidase method (Beckman Instruments, Fullerton, California, United States). Insulin was determined by radioimmunoassay (Linco, St. Louis, Missouri, United States).

The mean concentration of glucose and insulin was calculated for each sample time point. The trapezoidal rule was used to calculate the integrated response over 30-min intervals from 30 to 180 min for each participant. The integrated response was divided by its time interval to compute mean concentrations. Plasma glucose and insulin levels during the clamps averaged 5.17 ± 0.10 mmol l ^−1^ and 474 ± 14 pmol l ^−1^, respectively. This was 97.7% ± 0.2% of the desired goal with a coefficient of variation of 5.2% ± 0.4% in all clamps. Glucose utilization (M) for the 120- to 180-min interval was calculated from the amount of glucose infused after correction for glucose equivalent space (glucose space correction).

#### Effect of treatment with the peroxisome proliferator-activated receptor-gamma agonist rosiglitazone on A-SAA.

Eight healthy, nondiabetic, overweight or obese participants (age 44.7 ± 9.1 y, BMI 30.8 ± 3.1 kg m ^−2^, mean ± SD) were recruited and treated with rosiglitazone 4 mg d ^−1^ for 4 wk, followed by 8 mg d ^−1^ for 8 wk. Fasting serum levels of A-SAA were measured before and at 12 wk into the rosiglitazone intervention. At the same time points, subcutaneous abdominal fat biopsies were obtained under local anesthesia for ex vivo studies of adipose A-SAA expression and secretion (described below).

### Adipose Tissue Fractionation and Microarray Analysis

For microarray analysis, human omental and subcutaneous adipose tissues were obtained from four women (two obese, two nonobese) undergoing semielective intra-abdominal surgery at the University of Maryland Medical Center. Isolated adipocytes and stromal-vascular cells (SVCs) were obtained by collagenase digestion (final concentration 2 mg of collagenase per gram of fat tissue) in Kreb Ringer bicarbonate buffer containing 4% albumin and 200 nM adenosine (KRB-A). After centrifugation at ˜200 *g* for 1–2 min, the medium below the floating adipocytes (containing the SVCs) was removed and subjected to centrifugation at 800 *g* for 5 min. The pelleted SVCs were resuspended in KRB-A and washed three times using the same procedure. The floating adipocytes were washed three additional times with KRB-A. RNA was extracted from adipocyte and SVC fractions, and microarray analysis was conducted using Affymetrix (Santa Clara, California, United States) human U133A chips according to the manufacturer's instructions.

### Northern Analysis

Human adipose tissue and liver specimens were purchased from the National Disease Research Interchange (Philadelphia, Pennsylvania, United States), and total RNAs were prepared with Trizol (Invitrogen, Carlsbad, California, United States) according to the manufacturer's instructions. All other RNAs were purchased from Clontech (Palo Alto, California, United States). Total RNA (15 μg) extracted from the specified mouse (C57BL) or human tissue was subjected to agarose gel electrophoresis and blotted onto Nylon membranes using standard methods. Human SAA2 cDNA corresponding to nucleotides 1–536 of BC020795, and murine SAA2 cDNA corresponding to nucleotides 1–565 of U60438, were used as probes. These probes are 97% (human) and 95% (mouse) identical to SAA1 sequence and thus would be expected to hybridize to both SAA1 and SAA2. By contrast, the mouse SAA2 probe was only 62% identical to SAA3 mRNA and thus would not be expected to hybridize to SAA3 mRNA under the stringent wash conditions used. The probes were random-labeled (Stratagene, La Jolla, California, United States) with ^32^P-dCTP, and hybridization was carried out at 65 °C in Rapid-Hyb buffer (Amersham Biosciences, Piscataway, New Jersey, United States). Blots were washed twice with 0.5× SSC/1% SDS at 65 °C (stringent wash), and visualized by PhosphoImager (Amersham Biosciences).

### RT-PCR Analysis

For semiquantitative RT-PCR analysis, reverse transcription was carried out in a reaction containing 1 μg of total RNA, poly-dT primer, and MMLV reverse transcriptase using the Advantage kit (Clontech, Palo Alto, California, United States). PCR was performed under conditions typically consisting of 28 cycles of 94 °C for 30 s, 55 °C for 30 s, and 72 °C for 60 s. For detection of human A-SAA mRNA in fractionated adipocytes and SVCs, primers 5′- GAGAGAAGCCAATTACATCGGC -3′ and 5′- AGTATTTCTCAGGCAGGCCAGC -3′, which fully match both SAA1 and SAA2, were used. In addition, human SAA1 and SAA2 mRNAs were quantitated individually by RT-PCR using a common forward primer 5′- ATGGGGCTCGGGACATGTGGAG -3′, which was paired with reverse primer 5′- AGTCCTCCGCACCATGGCCTGT -3′ (SAA1-specific) or 5′- AGTCCTCCGCACCATGGCCAAA -3′ (SAA2-specific). Human β-actin was amplified as a control with primers 5′- TTAATGTCACGCACGATTTCC -3′ and 5′- AGACCTTCAACACCCCAGCCA -3′. RT-PCR products were electrophoresed on a 1% agarose gel, stained with ethidium bromide, and visualized by UV transillumination.

The level of adipose A-SAA mRNA expression was more accurately quantitated by real-time PCR. Applied Biosystems (ABI, Foster City, California, United States) TaqMan PCR kits with commercially available assay-by-design primers were used on an ABI PRISM 7900 Sequence Detection System. The primers and probe for SAA match both SAA1 and SAA2 genes and therefore measure total A-SAA (SAA1 and SAA2) mRNA. Cyclophilin A mRNA was used as an internal standard. Threshold cycle (C _T_) values were obtained and relative gene expression was calculated using the formula (1/2) ^CT SAA − CT cyclophilin A^.

### SAA Secretion from Adipose Tissue

To examine the relationship between adipose A-SAA secretion and BMI, and regulation by weight loss and rosiglitazone treatment, adipose tissue fragments were obtained at biopsy in premenopausal women over a range of BMI values (26.8 ± 4.2 kg m ^−2^, mean ± SD, *n* = 16), in postmenopausal women subjected to weight loss ( *n* = 33), and in participants before and after 12 wk of rosiglitazone therapy ( *n* = 7). Adipose tissue fragments were incubated for 3 h in M199 medium containing 1% BSA, and the medium was collected and stored at −80 °C until analysis for A-SAA. Adipocyte size was determined by a photomicrographic method [ 22].

For ex vivo studies of the regulation of SAA secretion, adipose organ culture was performed as previously described [ 23]. In a sterile hood, fresh human subcutaneous adipose tissue was minced into 5- to 10-mg pieces, washed with warm sterile saline, and cultured with no hormones, 25 nM dexamethasone (American Pharmaceutical Partners, Schaumburg, Illinois, United States), 7 nM insulin (Novo Nordisk, Princeton, New Jersey, United States), or a combination of these hormones, with and without rosiglitazone (1 μM ) (GlaxoSmithKline, Philadelphia, Pennsylvania, United States). The culture medium was changed daily and A-SAA was assayed in the conditioned medium on day 2.

### Effect of SAA on Cytokine Production

Primary human coronary artery endothelial cells were purchased from Cambrex (Walkersville, Maryland, United States) and grown in endothelial cell basal medium-2 (EBM-2) supplemented with EGM-2 BulletKit. All experiments were conducted between the third to fifth subcultures. Human primary adipose SVCs from the subcutaneous depot were isolated as described as above from a normal female participant (BMI 27.5 kg m ^−2^) who underwent elective abdominal reconstructive surgery. The SVCs were cultured in complete EGM-2, and subcultures between the second to third subcultures were used. RAW264 monocytes ( ATCC , Manassas, Virginia, United States) were grown in RPMI1640 medium supplemented with 10% fetal bovine serum. These cells were seeded on six-well tissue culture plates at about 75% confluence and grown to 90%–95% confluence. The growth medium was replaced with supplement-free media (EBM-2 basal medium for human coronary artery endothelial cells and RPMI1640 for RAW264 monocytes). The cells were treated 1 h after the medium change with recombinant synthetic human apo-SAA (Peprotech, Rocky Hill, New Jersey, United States), or vehicle (PBS). The endotoxin level for this commercial preparation was less than 0.1 ng μg ^−1^ protein. The conditioned medium was collected 8 h after SAA treatment by centrifugation at 2,000 *g* for 5 min and frozen until use for cytokine analysis. To examine the effect of SAA on adiponectin secretion, minced adipose tissue was cultured with recombinant SAA (2.34 μg ml ^−1^), and the conditioned medium was collected from 24 to 48 h of incubation for adiponectin assay.

### Effect of SAA on Lipolysis

Minced adipose tissue samples were cultured with recombinant SAA at a final concentration of 2.34 μg ml ^−1^. After 24 h, medium was collected and glycerol was measured using a fluorometric assay [ 24] to assess changes in lipolysis in response to SAA. Data are presented as micromoles of glycerol per gram of adipose tissue in 24 h.

### Cytokine Analysis

Human A-SAA (BioSource, Camarillo, California, United States) and PAI-1 (American Diagnostica, Greenwich, Connecticut, United States) were measured with ELISA kits according to instructions of the manufacturers. The SAA ELISA kit detects only A-SAA (SAA1 and SAA2) and not SAA4. The intra- and interassay coefficients of variation were 5% and 8%, respectively. Human monocyte chemoattractant protein-1 (MCP-1), IL-6, and IL-8; and mouse TNF-α, MCP-1, and RANTES in tissue culture media were analyzed at the Cytokine Core Facility, University of Maryland School of Medicine with cytokine multiplex reagents (Upstate Biotechnology, Lake Placid, New York, United States) by Luminex 100 (Luminex Corporation, Austin, Texas, United States). Human serum IL-6 and TNF-α levels were measured by high sensitive Quantikine enzyme-linked immunosorbent assay (R&D Systems, Minneapolis, Minnesota, United States), and adiponectin levels were measured by radioimmunoassay (Linco, St. Charles, Michigan, United States). All samples were assayed in duplicate.

### Statistical Analysis

Results are expressed as mean ± standard error of the mean (SEM) unless otherwise specified. Variables that were not normally distributed were natural logarithm-transformed for analysis and back-transformed for presentation. The Student's two sample or paired *t* test was applied when appropriate, as specified in the figure legends. Significance of correlations between two variables was determined by the Spearman rank correlation coefficient. In order to control for relatedness among the Amish participants, variance components analysis as implemented in SOLAR [ 25] was used to assess the correlation between BMI and A-SAA levels in the larger set of 134 Amish individuals. Differences were considered to be significant at *p* < 0.05.

## Results

### Acute-Phase SAA Is Highly Expressed in Human Adipocytes

**Figure 1:**
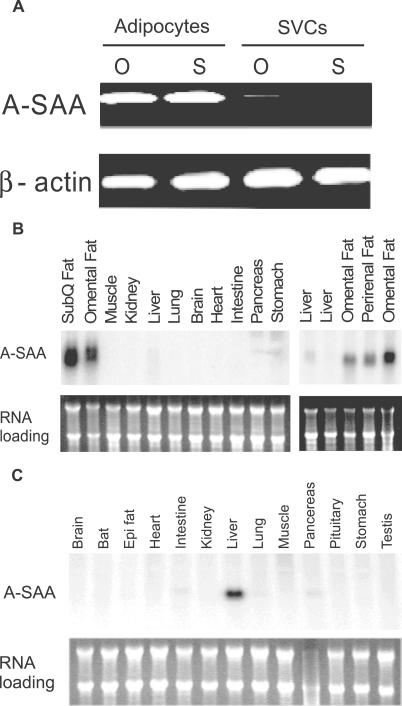
Tissue-Restricted Expression of A-SAA mRNA (A) Representative semiquantitative RT-PCR analysis of A-SAA and β-actin mRNA in SVCs and adipocytes fractionated from human omental (O) and subcutaneous (S) adipose tissues. (B and C) Northern analyses of multiple tissue blots from the human and mouse, respectively. For all Northern analyses, 15 μg of total RNA from the indicated tissues were electrophoresed, blotted onto a nylon membrane, and hybridized with a radiolabeled human (B) or murine (C) SAA2 cDNA probe, which detects both SAA1 and SAA2 (upper gels). Equality of RNA loadings was estimated by ethidium bromide staining (lower gels). Comparison of A-SAA expression was made in five independent participants (B, right). Epi, epididymal; SubQ, subcutaneous

Microarray analysis of mRNA preferentially expressed in fat cells compared to stromal cells of human subcutaneous and omental adipose tissue pointed our attention to A-SAA (unpublished data). Semiquantitative RT-PCR analysis with primers fully and specifically matching SAA1 and SAA2 cDNAs validated the high level of expression of A-SAA mRNA in human adipocytes but not in SVCs ( Figure 1A). Northern analysis showed that A-SAA mRNA was selectively and abundantly expressed in human adipose tissue ( Figure 1B, left). The higher expression of A-SAA mRNA in human adipose compared to liver tissue (at least 15-fold) was confirmed in an independent Northern analysis containing additional specimens of adipose and liver tissues ( Figure 1B, right). Conversely, in mice, A-SAA mRNA was predominately expressed in liver but not in adipose tissue ( Figure 1C). These studies show, unexpectedly, that in humans, A-SAA is predominately expressed in adipose tissue, more specifically in adipocytes, and that the adipose expression is species-specific.

### Obesity Is Associated with Increased Circulating Levels of A-SAA due to Increased Adipose A-SAA Gene Expression and Secretion

**Figure 2:**
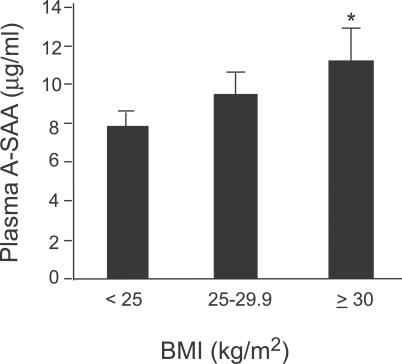
Circulating A-SAA Levels are Positively Correlated with BMI A-SAA levels were measured in plasma of normal human participants who were divided into lean (BMI < 25 kg m ^−2^, *n* = 54), overweight (BMI 25–30 kg m ^−2^, *n* = 49) and obese (BMI ≥ 30 kg m ^−2^, *n* = 31) groups. Data are expressed as mean ± SEM (ln-transformed for analysis, back-transformed for presentation), adjusted for age, sex, and family structure. * *p* = 0.013 versus lean group.

Selective and abundant A-SAA expression in adipocytes suggests that obesity may be associated with increased circulating A-SAA levels. To test this hypothesis, we measured plasma A-SAA levels in 19 age- and sex-matched nondiabetic sibling pairs who were discordant (>3 kg m ^−2^) for BMI. A paired t-test showed significantly higher plasma A-SAA levels in the heavier siblings ( *p* = 0.044) and a positive Spearman correlation coefficient was observed between the BMI and A-SAA differences ( *r* = 0.54, *p* = 0.017). In an expanded set of 134 nondiabetic men and women over a range of BMIs, BMI was a significant predictor of A-SAA level ( *p* = 0.025, controlling for age, sex, and family structure). When individuals were grouped ( Figure 2) into lean (BMI < 25 kg m ^−2^), overweight (25 kg m ^−2^ ≤ BMI < 30 kg m ^−2^), and obese (BMI ≥ 30 kg m ^−2^), the mean plasma A-SAA level of the obese group (ln-transformed for analysis, back-transformed for presentation) was 43% higher than that of the lean group ( *p* = 0.013, adjusted for age, sex and family structure).

**Figure 3:**
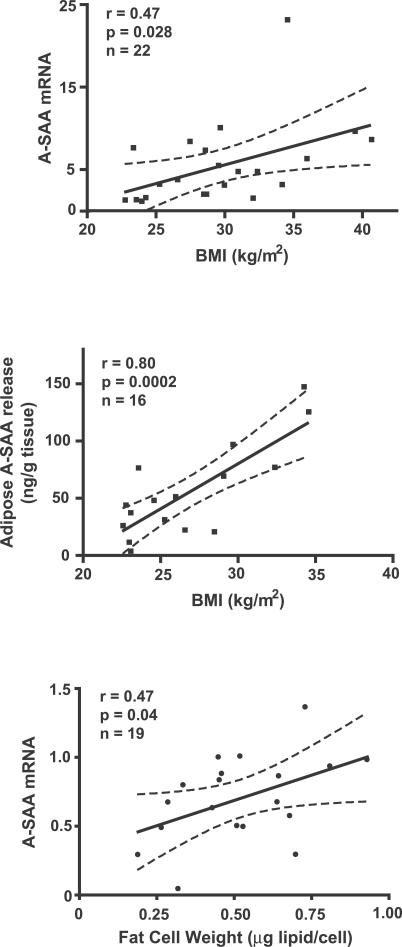
Adipose A-SAA Gene Expression and Secretion Are Increased with BMI Adipose A-SAA mRNA levels, measured by quantitative real-time RT-PCR (top) and A-SAA release by adipose tissue (middle), were significantly correlated with BMI. Furthermore, adipose A-SAA mRNA levels were increased with the adipocyte size (bottom). Dotted lines indicate 95% confidence intervals.

Increased serum A-SAA levels in obesity could be the result of normal expression and secretion of A-SAA from an increased fat mass, and/or increased expression and secretion of A-SAA from dysfunctional adipose or other tissues. To distinguish between these possibilities, adipose tissue samples were obtained from healthy premenopausal women over a range of BMIs, and A-SAA mRNA expression and secretion were measured. Adipose tissue A-SAA mRNA levels were significantly correlated with BMI ( *r* = 0.47, *p* = 0.028, *n* = 22; Figure 3A). Moreover, SAA release per gram of adipose tissue was strongly correlated with BMI ( *r* = 0.80, *p* = 0.0002, *n* = 16; Figure 3B). Furthermore, there was a strong correlation between adipose A-SAA gene expression and secretion in seven of these individuals who had both A-SAA mRNA and A-SAA secretion measured ( *r* = 0.89, *p* = 0.007, *n* = 7). A-SAA gene expression was also positively correlated with average adipocyte size ( *r* = 0.47, *p* = 0.04, *n* = 19; Figure 3C). These data suggest that increased A-SAA secretion from adipose tissue in obesity is the result of both increased fat mass and an increased rate of secretion per unit of adipose tissue.

### Changes in A-SAA Levels Are Associated with Outcomes of Clinical Interventions: Effect of Weight Loss and Rosiglitazone Therapy

**Figure 4:**
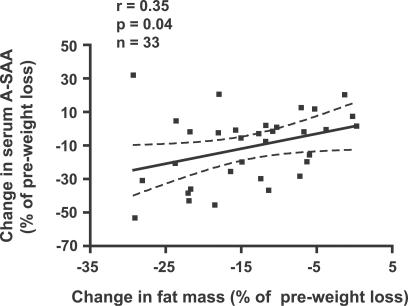
Reductions of Serum A-SAA and Fat Mass are Correlated Correlation between changes in serum A-SAA levels and changes in body fat mass before and after weight loss. Dotted lines indicate 95% confidence intervals.

If A-SAA is a marker of or causal link between obesity and its metabolic and cardiovascular complications, we would predict that circulating A-SAA levels would decrease in response to interventions that decrease obesity or its metabolic complications, e.g., insulin resistance. We measured serum A-SAA levels before and after weight loss with a hypocaloric diet program in 33 obese (BMI 32.3 ± 4.0 kg m ^−2^; mean ± SD) postmenopausal women. A mean (± SEM) weight loss of 6.0 ± 0.7 kg or 7.1% was associated with a 13.8% reduction in SAA levels ( *p* = 0.006, *n* = 33; paired *t*-test). Significantly, the relative changes in serum A-SAA concentration correlated with relative changes in BMI ( *r* = 0.39, *p* = 0.03, *n* = 33) and body fat mass ( *r* = 0.35, *p* = 0.04) ( Figure 4), but not with changes of fat free mass ( *r* = 0.23, *p* = 0.30). Eight of these participants also underwent hyperinsulinemic-euglycemic clamps before and after the weight loss intervention. In response to weight loss, increases in insulin sensitivity were correlated with decreases in A-SAA ( *r* = −0.74, *p* = 0.034). These findings suggest that A-SAA falls in response to weight loss and that a decrease in A-SAA partially predicts the increase in insulin sensitivity seen with weight loss in obese postmenopausal women.

**Figure 5:**
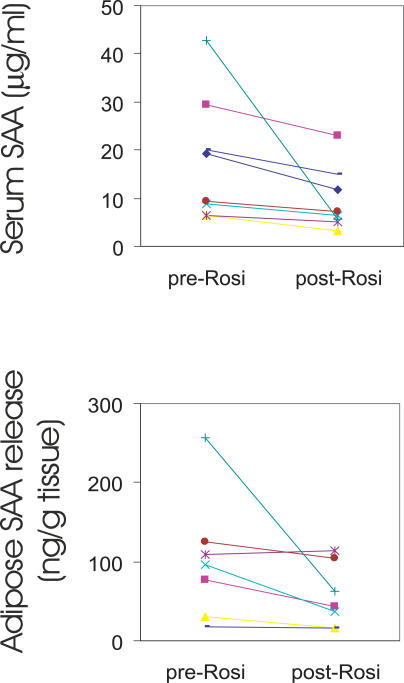
Rosiglitazone Reduces Serum A-SAA Levels and Adipose A-SAA Production in Humans Serum A-SAA ( *n* = 8) (top) and adipose secretion of A-SAA ex vivo ( *n* = 7) (bottom) were measured in nondiabetic participants before and after 3 mo of rosiglitazone treatment. The data are plotted with lines connecting the A-SAA levels of each individual. Serum A-SAA and adipose secretion of A-SAA (one symbol for same person of both studies) were significantly decreased by rosiglitazone ( *p* = 0.033 and *p* = 0.034, respectively; paired *t*-test after log-transformation).

We next reasoned that if A-SAA was an inflammatory adipokine involved in the metabolic consequences of obesity, other interventions that influence inflammation and insulin sensitivity might alter A-SAA concentrations. Eight overweight or obese nondiabetic individuals (BMI 30.8 ± 3.1 kg m ^−2^, mean ± SD) were treated for 12 wk with rosiglitazone, a drug with insulin sensitizing and anti-inflammatory actions. There were no statistically significant changes in body weight or fat mass in these individuals during the 12-wk intervention. Nevertheless, serum A-SAA levels decreased by a mean of 37% after treatment ( *p* = 0.033) ( Figure 5A). Moreover, the secretion of A-SAA from adipose tissue explants obtained by aspiration from these same participants was significantly reduced after rosiglitazone treatment ( Figure 5B). The extent of serum A-SAA decrease tended to correlate with that of adipose A-SAA secretion, although the correlation was not statistically significant, presumably due to the small sample size. Notably, one participant (green line in Figure 5) responded to rosiglitazone with a marked reduction in A-SAA. Exclusion of this individual changed the *p*-values for serum and adipose SAA secretion from 0.033 and 0.034 to 0.001 and 0.055, respectively. Thus, rosiglitazone reduced adipose A-SAA secretion and lowered serum A-SAA levels with no significant change in BMI or fat mass.

**Figure 6:**
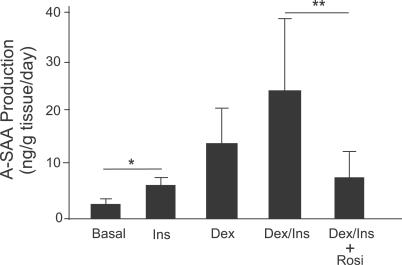
Rosiglitazone Directly Suppresses A-SAA Production in Adipose Tissue Human adipose tissue explants were incubated in cell culture medium 199 (basal) or medium with insulin (Ins, 7 nM) and dexamethasone (Dex, 25 nM) in the presence or absence of rosiglitazone (Rosi, 1 μM) for 48 h. A-SAA production between 24 and 48 h was measured and corrected for tissue weight. Data are expressed as mean ± SEM, *n* = 3 independent experiments. * *p* = 0.03, ** *p* = 0.002, two sample *t*-test after log-transformation.

The mechanism by which rosiglitazone decreases adipose A-SAA secretion could be direct, through action on adipose tissue, or indirect, through its effects on circulating hormones or other factors. Thus, we further investigated whether rosiglitazone acted directly on adipose tissue to decrease A-SAA secretion. Adipose tissue obtained from nondiabetic participants was cultured ex vivo. Incubation of the fat explants for 2 d with insulin or dexamethasone resulted in an SAA accumulation in the medium, and combination of the two hormones was additive in the stimulatory effect. Addition of rosiglitazone in the presence of insulin and dexamethasone reduced A-SAA secretion into the medium by 70% ( *p* = 0.002) ( Figure 6). These findings indicate that rosiglitazone acts directly on adipose tissue to suppress A-SAA production by adipocytes.

### SAA Is a Proinflammatory Cytokine

**Figure 7:**
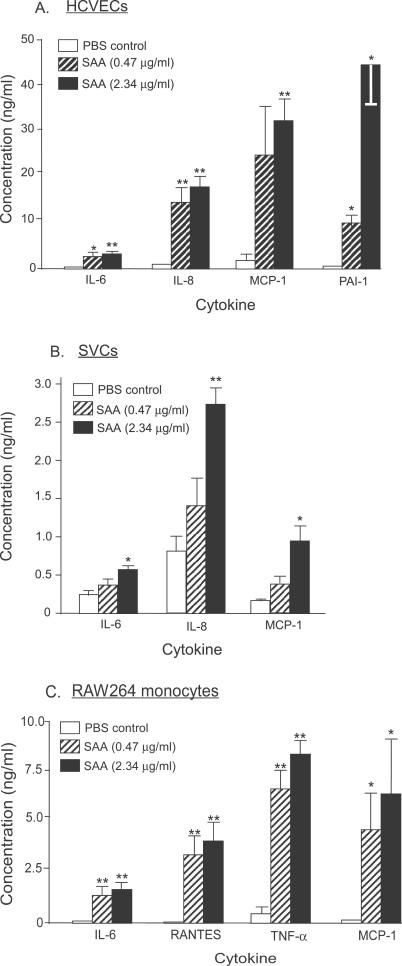
SAA Is a Potent Proinflammatory Mediator Human coronary artery endothelial cells (HCAECs, A), adipose stromal vascular cells (SVCs, B) and mouse RAW264 monocytes (C) were treated with vehicle (PBS, white bar), low (0.47 μg ml ^−1^, hatched bar), or high (2.34 μg ml ^−1^, black bar) concentrations of recombinant human SAA for 8 h in serum-free medium. Cell-free supernatants were then assayed for cytokines. Data are expressed as mean ± SEM from *n* = 3–5 independent experiments. Statistical significance (* *p* < 0.05; ** *p* < 0.01; two sample *t*-test) was observed between the SAA-treated groups and vehicle.

We hypothesized that A-SAA, produced by adipocytes, may be a causal link between obesity, chronic systemic inflammation, and metabolic and cardiovascular consequences through stimulation of inflammatory cytokines locally in adipose tissue as well as at distant sites. Primary human coronary vascular endothelial cells (HCVECs), adipose SVCs and mouse RAW264 monocytes were treated with vehicle (PBS), or with low (0.47 μg ml ^−1^) or high (2.34 μg ml ^−1^) concentrations of SAA for 8 h, and the conditioned medium was assayed for cytokine production. SAA dramatically stimulated, in a dose-dependent manner, the release of IL-6, IL-8, MCP-1, and PAI-1 in HCVECs; IL-6, IL-8, and MCP-1 in adipose SVCs; and IL-6, RANTES, TNF-α, and MCP-1 in RAW264 monocytes ( Figure 7). Treatment of these cells with 1 ng ml ^−1^ lipopolysaccharide, a concentration that is at least ten times higher than the maximum possible contamination of endotoxin in the recombinant SAA, did not stimulate inflammatory cytokine secretion in HCVECs or adipose SVCs. Thus, SAA is a potent proinflammatory adipokine.

### SAA Stimulates Lipolysis

**Figure 8:**
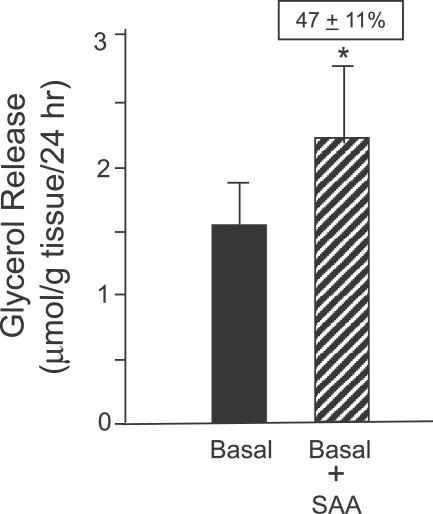
SAA Stimulates Lipolysis Adipose tissues (eight subcutaneous and one omental) were cultured in the presence or absence of SAA (2.34 μg ml ^−1^) for 24 h. SAA treatment increased lipolysis by 47% ± 11% as assessed by measurement of glycerol accumulation in the culture medium. Data are expressed as mean ± SEM (log-transformed for analysis, back-transformed for presentation). * *p* = 0.001, *n* = 9.

One mechanism by which obesity may be linked to insulin resistance is through increased lipolysis, which results in increased circulating levels of free fatty acids (FFAs) and decreased glucose uptake by muscle and liver [ 26]. Because chronic treatment with inflammatory adipokines such as TNF-α [ 27] and IL-6 [ 28] increase basal lipolysis, we next examined whether SAA might have a similar effect. Culture of human adipose tissue treated with SAA for 24 h significantly increased lipolysis, as measured by glycerol accumulation in the incubation medium by 47% ± 11% (mean ± SEM, *p* = 0.001) ( Figure 8).

### SAA Versus Adiponectin, IL-6, and TNF-α

Another mechanism by which adipose secretion of SAA might link obesity with insulin resistance is through down-regulation of adiponectin expression and/or secretion. To test this hypothesis, we treated human adipose tissue explants with SAA (2.34 μg ml ^−1^) and measured adiponectin secretion into the medium. We found that SAA tended to reduce adiponectin secretion, but the difference did not reach statistical significance (basal versus SAA treatment [ng g tissue ^−1^ 24 h ^−1^], 2.80 ± 1.6 versus 2.34 ± 1.27, *p* = 0.07, *n* = 9]. We further examined whether the levels of A-SAA in plasma of human participants over a wide range of BMIs were correlated with those of adiponectin. Although adiponectin levels were negatively correlated with BMI ( *r* = −0.3, *p* < 0.0001, *n* = 157), and SAA levels were correlated with BMI (see above), there was no correlation between the levels of A-SAA and adiponectin ( *r* = 0.049, *p* = 0.45, *n* = 157). These findings do not support a role of adiponectin in SAA-mediated pathways of insulin resistance.

We further examined the relationship of serum A-SAA levels with IL-6 and TNF-α in a population of postmenopausal obese women. Serum A-SAA is positively correlated with serum IL-6 ( *r* = 0.54, *p* = 0.03, *n* = 16), but not with serum TNF-α ( *r* = −0.30, *p* = 0.11, *n* = 30), which is consistent with the observation that there is no correlation between serum IL-6 and TNF-α levels ( *r* = 0.03, *p* = 0.93, *n* = 16). This finding suggests that common mechanisms may regulate A-SAA and IL-6.

## Discussion

Increasing evidence supports the hypothesis that the low-grade chronic systemic inflammation associated with obesity may be an important mediator of the metabolic syndrome and its constituents, including insulin resistance, type 2 diabetes, dyslipidemia, and hypertension [ 29– 31]. However, the molecular and cellular mechanisms that link obesity to inflammation are poorly understood. *SAA* is a multigene family consisting of four genes *(SAA1–4)* that are conserved in major vertebrates [ 32]. In humans, three of the four genes *(SAA1, SAA2,* and *SAA4),* but not *SAA3* (a pseudogene), are expressed [ 33]. In response to acute inflammatory stimuli, SAA1 and SAA2 levels in plasma can increase as much as 1,000-fold within 5–6 h and therefore, SAA1 and SAA2 are collectively known as acute-phase SAA (A-SAA) [ 34]. As with other acute-phase reactants, e.g., C-reactive protein, and based on animal studies, the liver is thought to be the primary source of circulating A-SAA [ 35].

In this study, we demonstrate that in humans, A-SAA (both SAA1 and SAA2) is predominantly expressed in adipose tissue, specifically adipocytes. Others have recently reported similar findings [ 13, 14]. Adipose expression of A-SAA in humans is in sharp contrast to mice, in which A-SAA is expressed predominantly in liver. There is a report of the expression of murine *SAA3* in adipose tissue [ 36], but this gene is not expressed in humans [ 33]. Similar to the results of other investigators [ 15, 16], we found that circulating A-SAA levels are elevated in obese compare to lean individuals and, furthermore, that A-SAA expression is correlated with BMI and fat cell size. Collectively, these results strongly support adipose SAA as a major source of circulating SAA, particularly in obese individuals.

Is A-SAA a marker of excess and/or dysfunctional adipose tissue and inflammation or is it a causal link between obesity, inflammation, and metabolic and cardiovascular sequelae? We demonstrated that interventions that are known to decrease chronic inflammation and improve the metabolic and cardiovascular consequences of obesity, such as weight loss and rosiglitazone treatment, also decrease adipose A-SAA expression and secretion as well as circulating A-SAA levels. Similar findings with regard to serum A-SAA levels were recently reported in persons who underwent weight loss [ 13, 14, 37], and in those treated with rosiglitazone [ 38]. Furthermore, previous studies indicated that A-SAA is a potent stimulus for the expression and release of TNF-α, IL-6, and IL-8 in neutrophils [ 17, 18, 39]. We showed also that A-SAA directly stimulates the production of inflammatory cytokines in coronary artery endothelial cells and monocytes, as well as locally by adipose tissue SVCs. Others have shown that A-SAA is induced by TNF-α and IL-6 in hepatoma cells [ 40], suggesting positive feedback between A-SAA and other cytokines. Collectively, these findings implicate A-SAA as a local and systemic proinflammatory adipokine, and not just a marker of inflammation.

**Figure 9:**
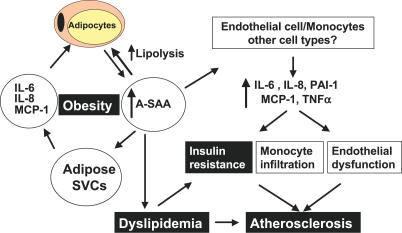
Schematic Diagram of Proposed Pathophysiological Role of Adipocyte-Derived A-SAA in Human Obesity A-SAA secreted from adipocytes acts locally on adipose SVCs to stimulate cytokine release and in adipocytes to stimulate lipolysis, increasing FFA release and decreasing insulin sensitivity in adipocytes, and possibly contributing to systemic dyslipidemia. In addition, A-SAA secretion by adipocytes into the circulation stimulates cytokine production at more distant sites, including in endothelial cells and monocytes, resulting in endothelial dysfunction, monocyte infiltration, accelerated atherosclerosis, and possibly insulin resistance in muscle and liver. A-SAA-stimulated lipolysis increases circulating FFA concentrations, further contributing to insulin resistance in muscle and liver. Finally, A-SAA incorporation into HDL accelerates its degradation and impairs its function, resulting in decreased HDL and accelerated atherosclerosis.

The increased mass of dysfunctional adipose tissue in obesity is known to be a source of several inflammatory factors, including TNF-α [ 41], IL-6 [ 42], and MCP-1 [ 43], and also of the prothrombotic factor PAI-1 [ 44]. These proinflammatory cytokines are predominantly products of SVCs within adipose tissue [ 42, 45]. By contrast, A-SAA, like leptin and adiponectin, is a product of adipocytes and not SVCs ( Figure 1A). Our finding that SAA potently stimulates the secretion of proinflammatory cytokines in adipose SVCs suggests that adipocyte A-SAA acts as a paracrine factor to enhance cytokine production by SVCs. In addition, obesity is associated with increased infiltration of adipose tissue by macrophages [ 10, 11], which may also be target cells of SAA action. SAA may also be a chemoattractant for macrophages, raising a possible link for the association of fat cell size with macrophage infiltration in obese adipose tissue [ 10]. Thus, A-SAA may act locally to alter cytokine production and fat metabolism as well as systemically on liver, muscle, cells of the immune system, and the vasculature, to impact insulin resistance and atherosclerosis ( Figure 9).

The signaling pathways of the A-SAA-mediated inflammation response are not well studied. In neutrophils, SAA induces IL-8 production through the formyl peptide receptor-like 1/lipoxin A4 receptor and activates nuclear factor kappa B [ 46]. The same signaling pathway recently has been shown to be an important mediator of inflammation-associated insulin resistance [ 47, 48]. Whether the induction of cytokine production by A-SAA in vascular endothelial cells and SVCs, as we have shown here, occurs through the same mechanism remains to be determined.

Our findings that rosiglitazone treatment significantly reduces A-SAA secretion and serum A-SAA levels suggest that *SAA1* and *SAA2* may be target genes of peroxisome proliferator-activated receptor-gamma. Both in vitro and in clinical studies, rosiglitazone exhibits anti-inflammatory properties [ 38, 49, 50], which are likely to be beneficial in slowing or reversing atherosclerosis [ 51]. Thus, the suppression of A-SAA may be a significant component of the anti-inflammatory and antiatherogenic action of peroxisome proliferator-activated receptor-gamma agonists, providing evidence that agents that inhibit A-SAA secretion or action may be efficacious for treatment of the metabolic syndrome and atherosclerosis.

Obesity is associated with increased basal lipolysis in adipose tissue [ 26] and elevated circulating FFAs that are thought to elicit systemic insulin resistance [ 52]. We also discovered that SAA, like IL6 [ 28] and TNF [ 27], has a long-term effect in stimulating basal lipolysis. The lipolytic activity of SAA can be an autocrine feedback mechanism by which increased SAA production ( Figure 3) from enlarged adipocytes limits further triacylglycerol accumulation. The resulting increased release of FFA into the circulation may contribute to insulin resistance. The mechanism of SAA-mediated lipolysis is under investigation. A recent study indicated that SAA binds CLA-1 (CD36 and LIMPII Analogous-1), resulting in activation of extracellular signal-regulated kinase 1/2 and p38 mitogen-activated protein kinases [ 53]. Extracellular signal-regulated kinase activation is involved in TNF-α-induced lipolysis [ 27]. CLA-1 is highly expressed in adipose tissue (unpublished data). Thus, SAA may act through CLA-1 and the extracellular signal-regulated kinase signaling pathway to stimulate lipolysis directly. Alternatively, increased lipolysis by SAA might be indirect, through its stimulation of other lipolytic cytokines, e.g., IL-6 and TNF-α.

Recent studies suggest that in addition to its role in inflammation, A-SAA may play a direct physiological role in cholesterol metabolism. SAA is an apolipoprotein and a component of high-density lipoprotein (HDL) particles [ 54]. The interaction of SAA with HDL may impair the function of HDL as an antiatherogenic molecule [ 55] and facilitate its degradation [ 56]. Dyslipidemia, including low HDL-cholesterol (HDL-C), is a metabolic consequence of obesity and a component of the metabolic syndrome [ 29]. Thus, the increase of adipose-derived SAA in obesity that we observed may be a mechanistic link between obesity, low HDL-C, and increased CVD risk ( Figure 9).

CRP is an acute-phase reactant produced in the liver and a marker of chronic low-grade inflammation. Modest elevation in CRP is associated with increased CVD risk in epidemiological studies [ 5, 57, 58]. In contrast to A-SAA, CRP is barely expressed in adipose tissue in humans (unpublished data). If A-SAA is a direct mediator of obesity-associated inflammation and its metabolic and cardiovascular consequences, might serum A-SAA be a better indicator of obesity-associated CVD risk than CRP? Ridker et al. [ 5] showed that both CRP and A-SAA levels confer similar risk for CVD events in participants of the Women's Health Study. Similarly, in the Women's Ischemia Syndrome Evaluation (WISE) Study, Johnson et al. [ 7] recently reported that SAA is independently associated with angiographic coronary artery disease and highly predictive of 3-y cardiovascular events. These studies did not specifically address the relationship between BMI and A-SAA (or CRP) in predicting CVD events. Our studies suggest that A-SAA may be a valuable diagnosticand prognostic marker of obesity-associated CVD risk and possibly of the effects of interventions such as weight loss and rosiglitazone therapy. Additional studies are needed to better define the biology and clinical utility of A-SAA and to further establish A-SAA as a causal link between obesity and inflammation and their cardiovascular consequences.

## Supporting Information

Alternative Language Abstract S1 Chinese Translation of the Abstract by RZY and DWG (21 KB DOC)

## Abbreviations

A-SAA: acute-phase serum amyloid protein A
BMI: body mass index
CRP: C-reactive protein
CVD: cardiovascular disease
FFA: free fatty acid
HDL: high-density lipoprotein
IL: interleukin
MCP-1: monocyte chemoattractant protein-1
PAI-1: plasminogen activator inhibitor-1
SD: standard deviation
SEM: standard error of the mean
SVC: stromal-vascular cell
TNF-α: tumor necrosis factor-alpha
