# Supplementary material for: Acute-Phase Serum Amyloid A: An Inflammatory Adipokine and Potential Link between Obesity and Its Metabolic Complications
Source: PLoS Med. 2006 Jun 6;3(6):e287. doi: 10.1371/journal.pmed.0030287 (PMC1472697; doi:10.1371/journal.pmed.0030287)
Supplement: Supplementary file 1 [file pmed.0030287.sg001.doc]

**Acute-phase Serum Amyloid A is a Pro-inflammatory Adipokine in**

**Humans and a Potential Link Between Obesity and its Metabolic**

**Complications**

Rong-Ze Yang^1^, Mi-Jeong Lee^2^, Hong Hu^1^, Toni I. Pollin^1^, Alice S. Ryan^2^, Barbara J.

Nicklas^3^, Soren Snitker^1^, Richard B. Horenstein^1^, Kristen Hull^1^, Nelson H. Goldberg^4^,

Andrew P. Goldberg^2^, Alan R. Shuldiner^1,2^, Susan K. Fried^2^ and Da-Wei Gong^1,5^

^1^Division of Endocrinology, Diabetes & Nutrition; ^2^Division of Gerontology, ^4^ Division

of Plastic and Reconstructive Surgery, University of Maryland School of Medicine at

Baltimore; Geriatric Research, Education and Clinical Center, Baltimore Veterans

Administration Medical Center; and ^3^Section on Gerontology and Geriatric Medicine,

Wake Forest University School of Medicine

Running Title: A-SAA, A Potential Link Between Obesity and its Metabolic

Complications

5 To whom correspondence should be addressed

660 W. Redwood Street, Room 497

Baltimore, MD 21201

Telephone: (410) 760-1672

Fax: (410) 706-1622

E-mail: dgong@medicine.umaryland.edu

人类急性时相血浆淀粉样蛋白A是一种联接肥胖症与其代谢性并发症之间的促炎症性脂肪细胞因子

***研究背景****：*研究表明肥胖症与机体轻度慢性炎症相关，而炎症反应的血清志标是发生心血管疾病的独立危险因素。然而肥胖与慢性炎症反应及心血管疾病相关的分子机制尚未清楚。***方法与结果****：*本研究测定了非肥胖及肥胖人群，肥胖症患者减肥前后及应用胰岛素增敏剂罗格列酮治疗前后急性时相血浆淀粉样蛋白A (A-SAA) 的血浆水平及其信使核糖核酸在脂肪组织中的表达水平和其在体外培养脂肪组织中的分泌水平。本文还检测了A-SAA在人类脂肪组织中血管基质细胞、冠状动脉血管内皮细胞及小鼠单核细胞株中的炎症诱导作用。结果发现A-SAA基因在人类脂肪细胞中有丰富且特异性地表达。脂肪组织中A-SAA信使核糖核酸表达水平及其分泌水平与体重指数相关 (相关系数r分别为0.47 和 0.80, p值分别为0.028 和 0.0002) 。肥胖症患者减肥后或罗格列酮治疗后血清A-SAA水平明显降低( p值分别为0.006及0.033) 。减肥后胰岛素敏感性改善幅度与血清A-SAA降低幅度有明显相关性(相关系数r为-0.74 , p值等于0.034) 。在SAA作用下，血管内皮细胞和单核细胞中白细胞介素6，白细胞介素8，肿瘤坏死因子α，单核细胞趋化蛋白1等炎症性细胞因子的产量增加了14 到259倍。此外， SAA还使培养的脂肪组织的基础脂肪分解作用增加47%。***结论***：人类A-SAA是一种促炎症性及促脂肪分解的脂肪细胞因子。肥胖症中脂肪细胞A-SAA表达增强提示它可能在局部和系统性炎症反应及游离脂肪酸的产生中起关键性作用。A-SAA很可能是肥胖与其并发症如胰岛素抵抗及动脉粥样硬化之间的直接纽带。因此，减肥和罗格列酮治疗所致的系统性炎症反应及胰岛素抵抗性的改善很可能部分是通过降低脂肪细胞的A-SAA的产生而介导的。

(翻译：杨荣泽，龚大为)
